# Sudden exposure to warm water causes instant behavioural responses indicative of nociception or pain in Atlantic salmon

**DOI:** 10.1016/j.vas.2019.100076

**Published:** 2019-09-27

**Authors:** Jonatan Nilsson, Lene Moltumyr, Angelico Madaro, Tore Sigmund Kristiansen, Siri Kristine Gåsnes, Cecilie Marie Mejdell, Kristine Gismervik, Lars Helge Stien

**Affiliations:** a Institute of Marine Research, P.O. Box 1870 Nordnes, NO-5817 Bergen, Norway; b Norwegian Veterinary Institute, P.O. Box 750, 0106 Oslo, Norway

**Keywords:** Behavioural response, Fish, Heated water, Nociception, Pain, Thermal delousing

## Abstract

Thermal treatment has become the most used delousing method in salmonid aquaculture. However, concerns have been raised about it being painful for the fish. We studied the behavioural response of Atlantic salmon acclimated to 8 °C when transferred to temperatures in the range 0–38 °C. Exposure time was 5 min or until they reached the endpoint of losing equilibrium and laying on their side, a sign of imminent death. At temperatures below 28 °C, none of the fish reached endpoint within the 5-min maximum. At 28 °C four of five fish reached endpoint, and fish reached endpoint more rapidly as temperature increased further. Fish transferred to temperatures above 28 °C had higher swimming speed immediately after transfer and maintained a high swimming speed until just before loss of equilibrium. Their behaviour was from the start characterised by collisions into tank walls and head shaking. Just before loss of equilibrium they started breaking the surface of the water, swimming in a circle pattern and in some instances displayed a side-wise bending of their body. In other words, salmon transferred to temperatures above 28 °C showed instant behavioural responses indicative of nociception or pain.

## 1 Introduction

As the salmon louse (*Lepeophtheirus salmonis*) has increasingly developed resistance against chemotherapeutants, thermal delousing by exposing fish to heated water (appr. 28–34 °C, sometimes higher) has become the most used delousing method in salmonid and especially Atlantic salmon (*Salmo salar* L.) aquaculture in Norway (Gismervik, Gåsnes, Nielsen, amp; Mejdell, 2018b; Overton et al., 2018). Thermal delousing is also used against *Caligus elongatus*, particularly in Northern Norway, and *Caligus rogercresseyi* in Chile (Sitjà-Bobadilla & Oitmann, 2017). Thermal delousing has been promoted as an environmentally friendly delousing method, as it only uses heated water. There is, however, often elevated mortality after thermal delousing compared to delousing the salmon with chemical baths or mechanical removal of lice by seawater flushing (Overton et al., 2018). Concerns have also recently been raised about thermal delousing being experienced as painful by the fish (Poppe, Dalum, Røislien, Nordgreen & Helgesen, 2018).

The ability of subjective experience, i.e. some level of consciousness or awareness, is by definition a prerequisite for experiencing pain IASP (1994), and there is an intense debate in the scientific literature on whether fish have this ability (see for example Key, 2016 and Sneddon et al., 2018 and the threads of responses to these articles). Absolute evidence for subjective experience in fish or other animals are obviously hard to obtain, and following the precautionary principle fish are included in European welfare legislation: EU directive 98/58/EC Article 2 includes ‘fish’ in the term ‘animals’, and Article 3 states that owners or keepers of animals must take all reasonable steps to ensure the welfare of animals under their care and to ensure that those animals are not caused any unnecessary pain, suffering or injury. The Norwegian Food Safety Authorities are therefore concerned that thermal delousing violates EU directive 98/58/EC and the Norwegian animal welfare act stating that keepers of animals, including fish, must ensure that the animals are treated well and are protected from danger of unnecessary stress and strains.

Fish have nociceptors for heat (Nordgreen et al., 2009; Sneddon, Brathwaite & Gentle, 2003), and salmonids exposed to warm water respond with abnormal behaviour such as jumps from the water, collisions and sudden swimming bursts (Elliott, 1991; Ineno, Tsuchida, Kanda & Watabe, 2005). From delousing operations in the industry, there are anecdotal reports about loud bangs and noises from within the treatment chambers, and flight reactions during treatment are suspected to be the cause of many of the injuries often seen (Gismervik et al., 2018a; Poppe et al., 2018). The water temperatures used for thermal delousing (28–34 °C) may be lethal within minutes (reviewed in Elliott & Elliott, 2010). The ultimate lethal temperature, i.e. the temperature at which fish cannot survive for 10 min, has been found to be 30–33 °C for Atlantic salmon parr and smolts (Elliott & Elliott, 2010). The much shorter exposure time of 30 s usually used during thermal delousing is however not likely to directly kill salmon, but tissue damage after treatment has been observed (Gismervik et al., 2018b; Poppe et al., 2018) although it is not known if this is due to temperature *per se* or other aspects of the treatment.

Coldwater is a new candidate method for thermal delousing (Overton et al., 2019). Live chilling induces stress in salmon (Roth, Slinde & Robb, 2006) and temperatures below 0 °C may be lethal (Elliott & Elliott, 2010). Elliott (1991) observed behavioural responses to rapidly decreasing temperatures similar to the responses seen for rapidly increasing temperatures. Thus, although no nociception has been documented at low temperatures (Ashley, Sneddon & McCrohan, 2007), sudden exposure to very low temperatures appear to be aversive to salmon.

In the present study, we aimed to investigate if there is a temperature threshold within the range from 0 to 38 °C from where salmon post-smolts transferred from 8 °C respond with clear behavioural changes indicative of nociception or pain. The experiment was conducted in accordance with Norwegian regulations on animal experimentation under permit number 15383.

## 2 Material and method

### 2.1 Subjects

The subject fish were out-of-season Atlantic salmon (*Salmo salar* L.) smolts of the AquaGen strain, hatched 16 January 2017 and transferred from freshwater to tanks (1.5 m wide, 1200 L) with seawater (salinity 34 ppt, temperature 8–9 °C) on 17 October 2017, held on a natural light regime and fed dry feed (Skretting Spirit Supreme) during daytime. Four weeks before the start of the experiment, on 6 April 2018, 125 fish were distributed into three tanks that were located next to the experimental tank to minimize air exposure time and handling during transfer to the experimental tank (see below for tank description). The fish were stocked at ~8 kg m^–3^, well within the 17 kg *m* ^−^ ^3^ upper limit for good welfare defined in the RSPCA welfare standards for farmed Atlantic salmon (RSPCA, 2018). The fish were starved for two days before the experiment started. The mean ± *S*.D. length and weight of the fish during the experiment were 279 ± 23 mm and 234 ± 52 g, respectively.

### 2.2 Experimental tank

The experimental tank where fish were exposed to seawater (34 ppt) of various temperatures (Table 1) was of the same type as the stock tanks: squared with rounded corners, 150 cm wide and light grey of colour, but the water depth in this tank was restricted to 15 cm depth (volume around 300 L) in order to facilitate temperature regulation. The lid of the tank was partly lifted and a camera (GoPro™, San Mateo, CA, USA) was attached to the lid so that it recorded the entire water volume from above. Temperature (Testo 176T2, Testo Ltd., UK) and oxygen (Handy Polaris 2, OxyGuard Inc., Denmark) were logged immediately before a new fish was transferred to the tank. Water temperature of 0 °C was obtained by adding blocks of frozen seawater to the tank. Remaining ice was removed before fish were transferred to the tank. Water temperature of 4 °C was thereafter obtained by adding seawater of ambient temperature (8 °C). For the control group, which was exposed to water of ambient temperature, i.e. exactly the same temperature as they were transferred from, all water was replaced before transfer of fish. For the remaining groups, which were exposed to higher temperatures than the ambient, water was regulated by adding heated seawater. The exposure temperature never deviated more than 0.5 °C from the group mean for any individual fish.

**Table 1:** Temperature groups, group sizes (n), measured exposure temperatures (mean ± *S*.D.) and oxygen saturation (mean ± *S*.D., percent of air saturation) in the experimental groups.

Group	n	Temperature ( °C)	O_2_ (%)
0	6	0.5 ± 0.3	86 ± 3
4	6	4.1 ± 0.1	95 ± 0
8	5	8.5 ± 0.0	99 ± 1
12	6	12.2 ± 0.2	100 ± 1
16	6	16.1 ± 0.2	99 ± 1
20	5	19.9 ± 0.2	100 ± 1
24	5	24.1 ± 0.2	109 ± 3
26	6	26.1 ± 0.2	101 ± 2
28	5	28.1 ± 0.3	108 ± 3
30	6	30.1 ± 0.2	102 ± 1
32	6	32.0 ± 0.2	99 ± 1
34	6	34.0 ± 0.2	100 ± 1
36	6	36.1 ± 0.2	97 ± 1
37	4	36.9 ± 0.1	95 ± 1
38	4	38.0 ± 0.1	96 ± 1

### 2.3 Humane endpoints

The endpoint for taking an individual out of the experiment was when it lost equilibrium and laid on the side for 2 s. Fish that reached this endpoint was immediately netted out and euthanized by and overdose of tricaine methanesulfonate (Finquel, Ayerst Laboratories, New York). The rational for subjecting fish for both longer and higher temperatures than the current industry standard was that we were uncertain if the acute response to transfer itself could be distinguished from the response to temperature within 30 s. Furthermore, it is important to study the “safety margins” in terms of temperature and exposure time, especially as higher temperatures and longer treatment times may be attempted if the salmon louse develop resistance to warm water (Ljungfeldt, Quintela, Besnier, Nilsen & Glover, 2017).

### 2.4 Procedure

Single fish were netted out of the holding tank and immediately released into the experimental tank. Each fish was left in the tank for maximum 5 min or until it reached the endpoint. The fish was then netted out and euthanized in an overdose of tricaine methanesulfonate (Finquel, Ayerst Laboratories, New York). Temperature was controlled and regulated by adding cold or warm seawater and then removing excess water (if needed) before a new fish was transferred. The behaviour of the fish was recorded on video by the GoPro camera. From 0 to 24 °C the temperature was increased with 4 °C for each new temperature group, and from 24 °C with 2 °C, in order to increase the resolution in the higher temperature interval where the temperature threshold was expected to be. Up to 36 °C each temperature group included six fish, but only four fish were exposed to 38 °C as we from subjective estimation of the behavioural response during exposure judged this temperature to be unacceptably high. Instead of increasing the temperature further, we exposed four fish to 37 °C to increase the resolution at high temperatures. For practical reasons with heating and cooling of the water the sequence of tests became: Day 1: 0, 4, 8, 20, 16, 12; Day 2: 28, 30, 32, 34, 36, 38; Day 3: 24, 26.

Four video files for the 0–36 °C groups were corrupt and could not be opened and viewed, probably due to camera malfunction or mistakes by the operator. The number of fish analysed in each temperature was therefore only 5 for some of the groups (Table 1). All four videos were analysed in each of the 37 and 38 °C groups (Table 1).

### 2.5 Analysis of behaviour

#### 2.5.1 Estimation of swimming speed

Swimming speed was estimated using a script created in MATLAB R2018b (The MathWorks, Inc., USA). Input to the script was which movie to analyse, and how many seconds since start of exposure the analysis should be performed. The script then opened the movie at the given time, and extracted four subsequent image frames, 1/3 of a second apart (0, 1/3, 2/3 and 3/3 into the second). The script then displayed the four images, and the position of the snout of the fish was manually marked using the mouse. Euclidian distance between these four consecutive points was then calculated as an estimation of swimming speed per second at the given time and normalised to the length of the fish in the images to standardise the speed into body length per second (BL s^–1^). Since this analysis was relatively laborious, it was done every second for the 10 first seconds of the exposures to get high resolution immediately after transfer, then every 5 s from 10 to 60 s of the exposures, every 10 s from 60 to 200 s of the exposures, and finally only every 20 s from 200 to 300 s of the exposures when behaviour had stabilised.

#### 2.5.2 Behavioural events

In addition to surface breaks and collisions as described by Elliot (1991), three random video clips from high temperatures (34–38 °C) was studied in order to find other easily countable abnormal behaviours, e.g. behaviours assumed to reflect responses to high temperature. These were then defined for use when behaviour of all temperature groups was analysed and are described in Table 2. Video clips showing examples of the behavioural categories are available in the online supplementary material. During these analyses, the temperature was blinded to the observer in order to avoid observation bias. An open source software (CowLog 3.0.2, Pastell, 2016) was used to analyse the videos for the behavioural categories. In this program, behavioural categories defined by the user are programmed with keyboard shortcuts that are pressed when a behaviour is observed. Frequency and latency of each behavioural event is then the output from the analysis.

**Table 2:** Behavioural events counted during video analyses.

Behaviour	Description
Direction change	The fish changes swimming direction with >90° within 1 body length
Circling	The fish swims in a circle (>270°)
Head shake	The fish makes rapid shakes with the head
Collision	Uncontrolled collision with the tank wall
Surface break	The fish breaks the surface with its head
Bend	The fish bends the body sideways >90° without moving forward

### 2.6 Statistics

Although some of the data could have been analysed using non-linear parametric methods, all data are analysed using the same non-parametric methods for consistency. The results are presented as median [25-, 75-percentile] observed time to endpoint, median [25-, 75-percentile] swimming speed, or median [25-, 75-percentile] frequency of behaviour event. Since the output data typically looked like step functions (Crawley, 2007), non-parametric conditional inference trees were used to objectively find thresholds and classify temperature intervals according to swimming speed and behaviour frequencies (R package party, function ctree, Hothorn, Hornik & Zeileis, 2006). Spearman rank correlation was used to test if the observed values increased or decreased with temperature (function cor.test, method = "spearman", R version 3.5, R Core Team 2018).

## 3 Results

### 3.1 Time to endpoint

**Fig. 1:**
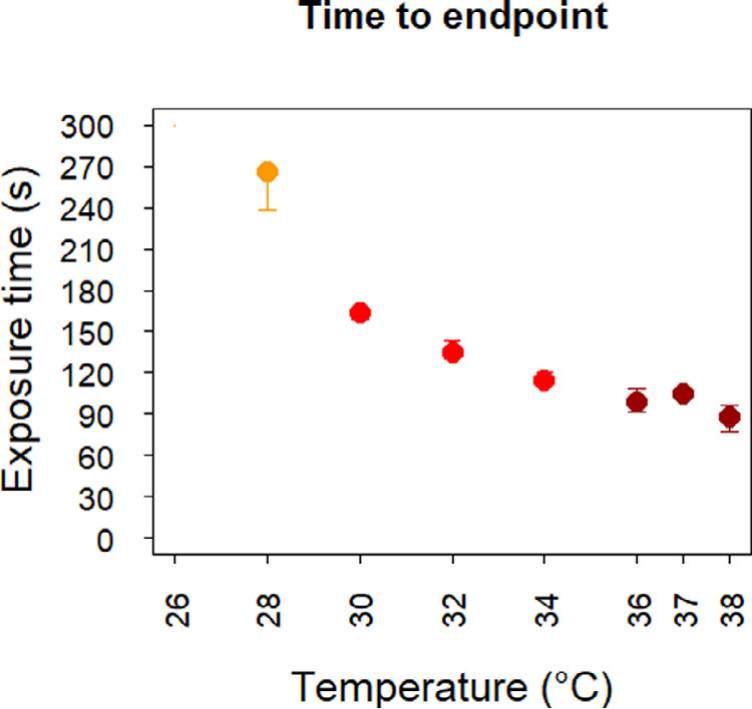
Median time to endpoint (laying motionless on the side for >2 s) at different temperatures. The error bars represent the 25- ant 75-percentiles. No fish reached the endpoint within the maximum exposure time of 300 s at temperatures below 28 °C. Point colouring is given for easy identification of temperatures.

At 28 °C four of five fish reached endpoint (i.e. lost equilibrium and laid on the side for >2 s) before end of the pre-set maximum exposure time of 300 s. Median time of endpoint for these four fish was 251 [237, 266] s, or if including the fifth fish as a 300 s observation, the median endpoint was 266 [238, 267] s. While none of the fish exposed to <28 °C reached the endpoint, all the fish that were exposed to temperatures >28 °C did so, with time to endpoint decreasing with increasing temperature (r_s_ = 0.96, *p* = 0.002, Fig. 1).

### 3.2 Swimming speed

**Fig. 2:**
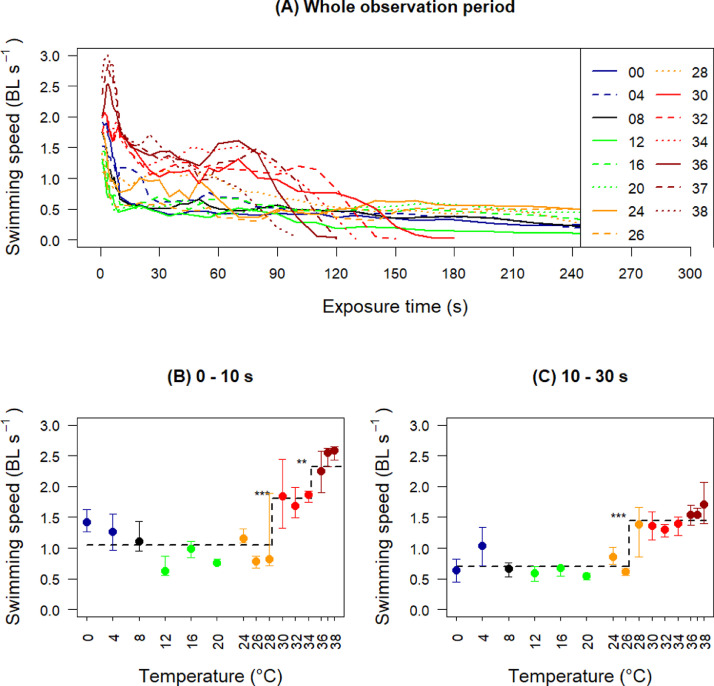
Swimming speed per temperature group. A) Measured swimming speeds from start of exposure till endpoint, or maximum 300 s of exposure. B) Median swimming speed first 10 s. C) Median swimming speed in the period 10–30 s after exposure. The error bars represent the 25- and 75-percentiles. *p < 0.05, **p < 0.01, ***p < 0.001 indicate significance of the thresholds found by the ctree-algorithm. Point colouring is given for easy identification of temperatures. (For interpretation of the references to colour in this figure legend, the reader is referred to the web version of this article.)

All temperature groups had an initial peak in swimming speed immediately after being released into the treatment tank (Fig. 2A). The ctree-algorithm identified two distinct increasing steps for initial swimming speed (first 10 s), one threshold between 28 and 30 °C (*p*<0.001), and one threshold between 34 and 36 °C (*p*<0.001) (Fig. 2B). After the first 10 s, the fishes in all groups reduced their swimming speed (Fig. 2A), but the fishes subjected to 28–38 °C maintained a higher swimming speed than the fishes subjected to the lower temperatures (Fig. 2C), until the speed dropped as they approached their endpoint (Fig. 2A and Fig. 1).

### 3.3 Behaviour events

**Fig. 3:**
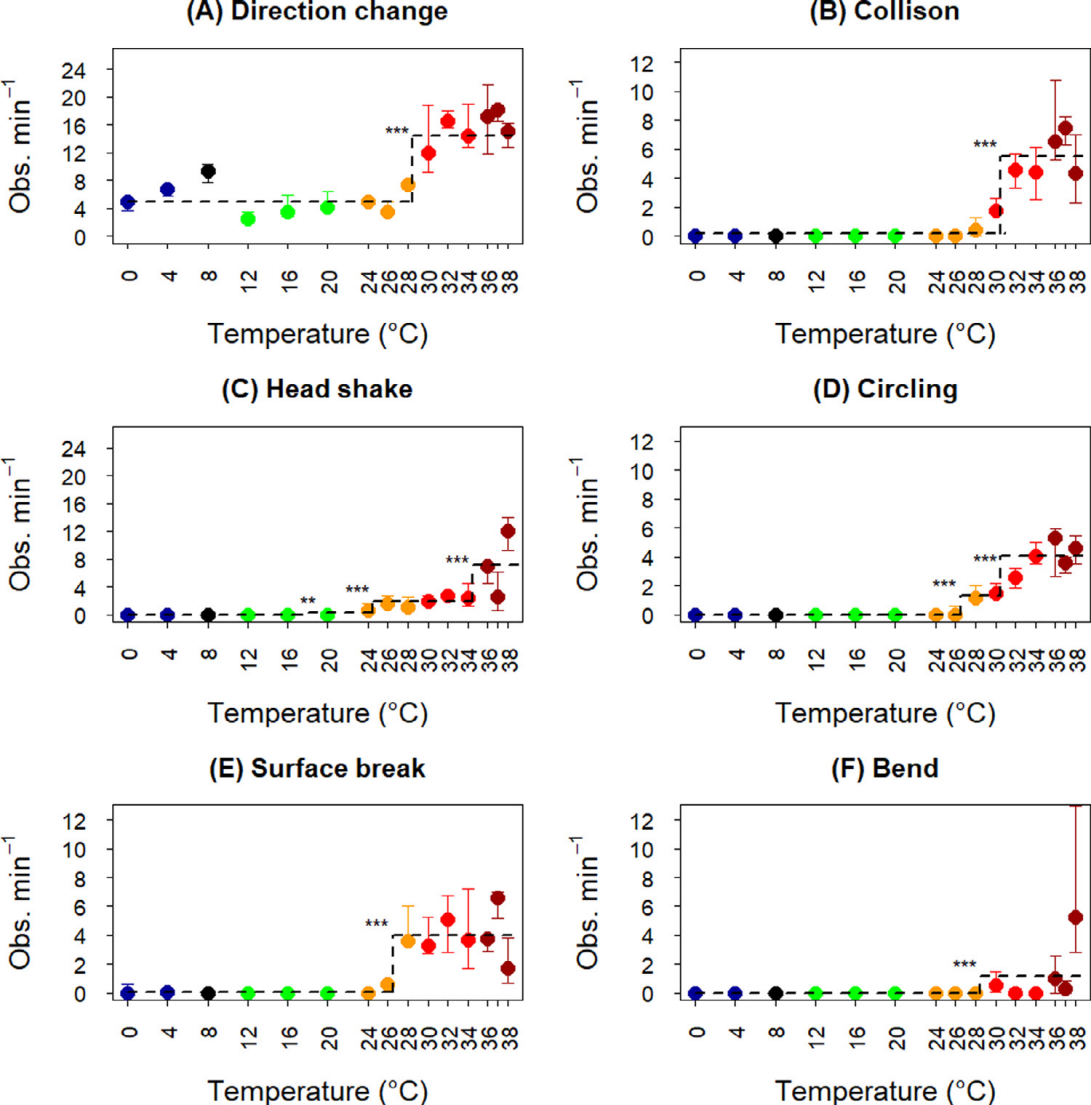
Median observed frequency of behavioural events per minute before end of exposure (300 s) or laying on the side for the fish in each temperature group 0–38 °C. A) Direction change, B) Collison, C) Head shake, D) Circling, E) Surface break, F) Bend. The error bars represent the 25- and 75-percentiles. *p < 0.05, **p < 0.01, ***p < 0.001 indicate significance of the thresholds found by the ctree-algorithm. Point colouring is given for easy identification of temperatures.

**Fig. 4:**
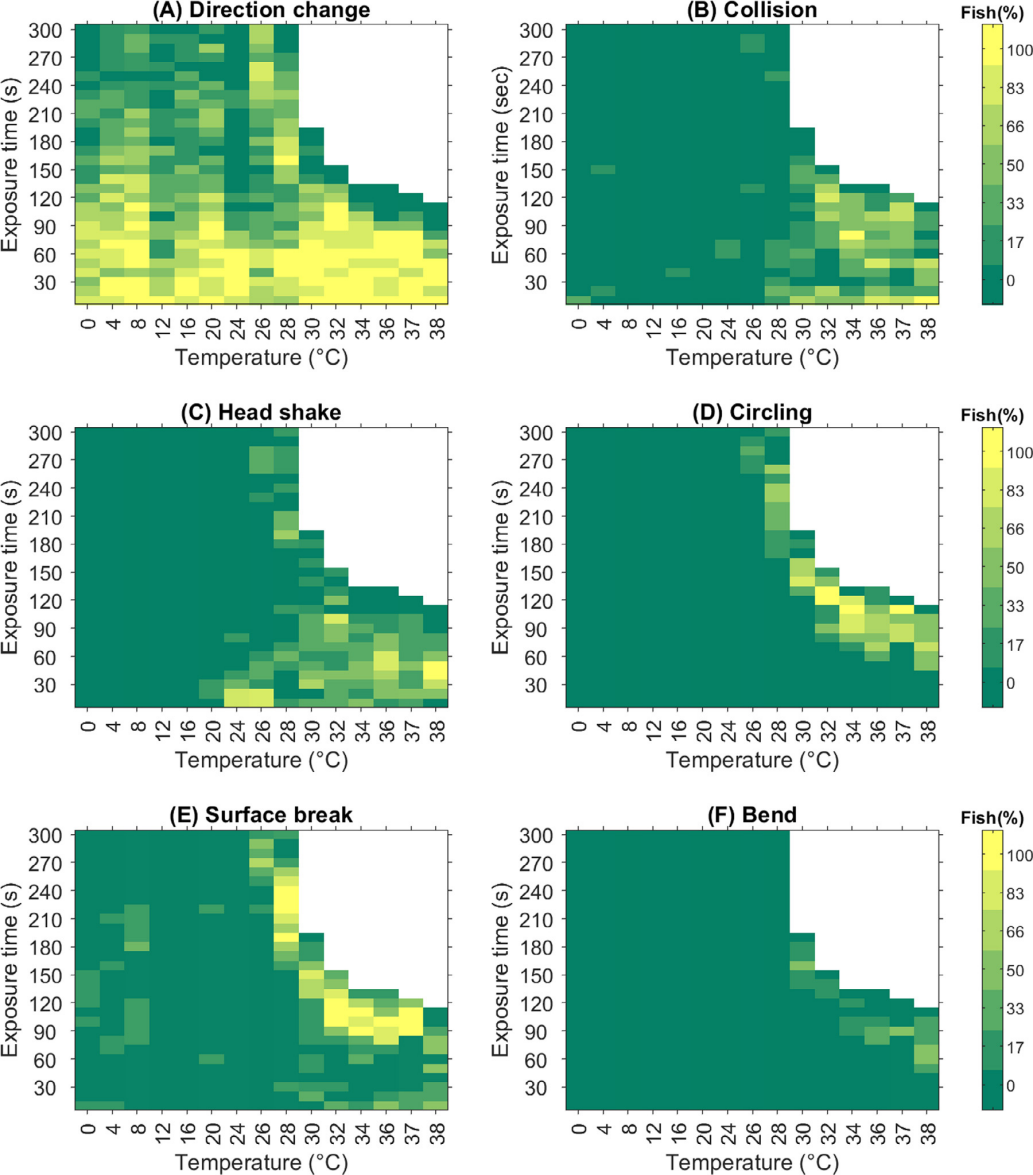
Behaviour map showing the proportion of fish at each exposure temperature performing a behavioural response in each 10-s interval of the observation period, as indicated by the colour scale. White fields indicate that all fish at that temperature have reach the endpoint. A) Direction change, B) Collison, C) Head shake, D) Circling, E) Surface break, F) Bend.

The ctree-algorithm identified a clear step in observed ‘Direction change’ per minute of observation time between 28 and 30 °C (*p*<0.001, Fig. 3A). However, ‘Direction change’ occurred frequently during the first 100 s of the exposure at all temperatures (Fig. 4A), but the lower frequency later in the observation period resulted in different “per minute of observation time” levels (Fig. 3A). ‘Collision’ mainly occurred above 30 °C, where the ctree-algorithm identified a clear step (*p*<0.001, Fig. 3B). However, directly comparing collisions at 28 °C with observed collisions for the controls gives significant more collisions compared to controls also for this relatively low temperature (0.00 [0.00, 0.00] vs. 0.45 [0.00, 1.26] obs. min^−1^, r_s_ = 0.64, *p* = 0.045). The fish started colliding immediately after transfer to exposure temperatures and continued to do so until they reached the endpoint (Fig. 4B). ‘Head shake’ was not observed below 20 °C, from where the ctree-algorithm identified a minor step (*p* = 0.004), and two more clear steps between 24 and 26 °C (*p*<0.001) and after 34 °C (*p*<0.001, Fig. 3C). At 20 and 24 °C the majority of the head shakes occurred during the first 30 s (Fig. 4C). At 26 and 28 °C head shakes occurred mainly in the beginning and the end of the observation period, while at higher temperatures they occurred throughout the observation period (Fig. 4C). ‘Circling’ was only observed at 26 °C or higher, with the highest rate at the highest temperatures (Fig. 3D), and usually started to occur shortly (~1 min) before the endpoint (Fig. 4D). Fish performing ‘Circling’ typically swam partly with the side facing upwards but still moved relatively fast. ‘Surface break’ occurred at highest rates at the higher temperatures, with a clear threshold between 26 and 28 °C (*p*<0.001, Fig. 3E), but there were also random occurrences at the lower temperatures (Fig. 4E). As with ‘Circling’, ‘Surface break’ was generally most frequent during the last minute before the fish reached their endpoint (Fig. 4E). ‘Bend’ was never observed at temperatures below 30 °C. In contrast, 47% of the fish from 30 °C and above performed ‘Bend’, but with high variation between individuals (1–35 bends) (Fig. 3F). ‘Bend’ typically occurred just before the fish reached the endpoint (Fig. 4F), with median occurrence of first ‘Bend’ 15.2 [9.5, 25.0] s before the endpoint.

## 4 Discussion

When transferred to the experimental tank salmon responded with elevated swimming speed and frequent changes of swimming direction at all temperatures, although the responses were stronger at the highest temperatures. However, while fish exposed to low and intermediate temperatures calmed down within half a minute, fish exposed to temperatures above 28 °C sustained a high swimming speed, showed behaviour such as ‘Collision’, ’Surface break’ and ‘Bend’, before they lost equilibrium and eventually stopped moving. The behavioural responses occurred earlier the higher the temperature.

To our knowledge this is the first study of salmon behaviour after direct transfer into high temperatures. Peterson and Anderson (1969) exposed salmon parr to rapid (~30 min) changes in temperature from 6 °C to maximum 21 °C, and from 18 °C to maximum 27 °C and minimum 6 °C. The fish responded during the temperature changes with marked increases in activity level, which then quickly died down once the temperature had stabilised, except for the fish on 28 °C which maintained high activity also after the change was complete. Elliott (1991) exposed salmon parr to temperatures up to 36 °C, but did so by gradually elevating the temperature 1 °C h^–1^ rather than by a sudden transfer as in the present study. The study by Elliott focused on thermal tolerance in terms of feed intake and survival and gives only a brief description of the behavioural responses. Still, the responses described by Elliott, which included ‘sudden bursts of activity with frequent collisions with the tank sides, rolling and pitching’, followed by ‘short bursts of weak swimming’, and the maintained high activity on 27 °C described by Peterson and Anderson, correspond well to the responses described in the present study. Elliott also noted that in the last phase before death, movements were restricted to the opercula, pectoral fins and eyes, but such detailed observations were not possible in the present study.

There was little response to cold water and no differences from the control (8 °C) was found. Nociceptors do not respond to low temperatures (Ashley et al., 2007), and strong behavioural responses were therefore not expected. Elliott (1991) reports that for salmon parr, thermal stress due to falling temperature resulted in sudden bursts of activity followed by a coma-like state. This coma-like state was also seen in the cold-water delousing experiment by Overton et al. (2019), but the temperature drop was here from 15 °C, and not from 8 °C as in the present study. In the present study, no fish lost equilibrium within 5 min at the lower temperatures, and swimming speed was classified as being in the same group as the control by the ctree-algorithm.

Temperature is one of the main drivers for spatial distribution of caged salmon, and salmon actively avoid temperatures above 18 °C (reviewed by Oppedal, Dempster & Stien, 2011). Still, signs of alarm in terms of frequent collisions, surface breaks and increased swimming speed were not significant when salmon were transferred directly from the 8 °C acclimation temperature to tanks holding up to 26 °C. At 28 °C initial median swimming speed did not differ from the control, but the variation was high with some individuals swimming fast, and the fish at 28 °C maintained high median swimming speed throughout. Also, the identified step thresholds for the frequencies of the different behavioural events varied to start between 26 and 28 °C (‘Circling’, ‘Surface break’), between 28 and 30 °C (‘Direction change’, ‘Bend’) or between 30 and 32 °C (‘Collisions’), except for ‘Head shake’ which started already at 20–24 °C. All in all, and also considering that comparing collisions at 28 °C revealed significant difference from the control temperature, makes it reasonable to set an overall thresholds for when temperature is acutely aversive around 28 °C. This was also the lowest temperature at which fish reached the endpoint within the 5-min observation period.

At the endpoint when a fish had stopped moving and lay on the side it was removed from the tank and euthanized, except for one individual that was returned to an 8 °C tank for several minutes. This fish did not regain any signs of motion and had no opercular movements, it was dead. Severe tissue damage, including gill- and brain bleeding, was found on a subset of fish that were examined for tissue damage after exposure to 34–38 °C (Gismervik et al., 2019). Although no systematic attempts were made to study the probability to survive after reaching the endpoint, we therefore assume this state to be life threatening. Elliot (1991) found, by elevating temperature relatively slowly (1 °C h^–1^), that 31 °C was the highest temperature at which salmon parr acclimated to 10 °C survived for 10 min, while fish in the present study reached the endpoint and were likely doomed within 5 min at 28 °C, and for 38 °C already within 1.5 min. With different methods and life stages in these two studies comparisons should be made with caution, but it is possible that the rapid onset of loss of equilibrium and probable death in the current experiment can be explained by the temperature shock at direct transfer to the exposure temperature. Fish size may also play a role for the temperature tolerance. Intuitively one might expect that larger fish, with a lower surface-area-to-volume ratio, are more resistant to high temperatures (Stevens & Fry, 1970). However, Huntsman (1942), studying thermal death of wild salmon in a river during hot summers, reported that at 29.5 °C the largest salmon died first, and that ‘of at least 12 grilse in the vicinity only 4 died and not a single parr’. Decreasing temperature tolerance with increasing size was subsequently confirmed experimentally (Huntsman, 1942).

Ashley et al. (2007) showed that mechanothermal nociceptors in rainbow trout had a thermal threshold at ~29 °C, while the polymodal nociceptors had a thermal threshold at ~33 °C. These receptors are in the skin, explaining the instant reaction to temperatures above 28 °C, before internal physiological processes can have been affected and caused behavioural impairment. The further increase in swimming speed identified between 34 and 36 °C seen in Fig 2B could reflect that also the polymodal receptors respond to the highest temperatures. The salmons’ behavioural reactions to high temperatures were instantaneous, not only with fast swimming, but also with collisions into tank walls and head shaking (Fig 3B and C, Fig 4B and C), suggesting a response with loss of control of their behaviour.

The temperature threshold for strong behavioural responses, i.e. 28–30 °C, corresponds with the lethal temperature threshold. A similar notion was made by Ashley et al. (2007) for rainbow trout (*Oncorhynchus mykiss*), where the heat threshold for nociceptors to respond (29–33 °C) was within the range of short-term lethal temperature of 30 °C (Ineno et al., 2005). It is therefore reasonable to assume that the behavioural responses to high temperatures reflect nociception, and that the nociceptors have evolved to respond at temperatures that may lead to tissue damage and ultimately death. Although Ashley et al. (2007) found the mean thresholds for the mechanothermal and the polymodal nociceptors at respectively ~29 °C and ~33 °C, they also report some measurements down to 20 °C for the polymodal and down to 22 °C for the mechanothermal. There were, however, little evidence that such early responses of nociceptors were intense enough to elicit significant behavioural responses in the current study.

‘Head shake’ was performed by no fish below 20 °C, by one individual at 20 °C and by most individuals at 24 °C and above. At 20 and 24 °C ‘Head shake’ was only performed early during the observation period, at 26 and 28 °C they were performed early and then subsided, and performed again late in the observation period, while at 30 °C and above ‘Head shake’ was performed throughout the period until they approach the endpoint. Observations of head shakes was difficult for fish with rapid swimming, surface breaks etc., and thus the occurrence of ‘Head shake’ was likely underestimate at the higher temperatures. It is difficult to know exactly why the fish were shaking their heads. Roberts, Johnson and Casten (2004) observed that rainbow trout with bullae on the gills from parasites would shake their heads as a response to the irritation. As the response subsided at 20–24 °C it may be that the fish initially experienced some irritation that was lost when they had overcome the transition shock, while at higher temperatures the irritation returned (26–28 °C) or remained (30 °C and above).

Shortly before the fish exposed to the higher temperatures reached the endpoint, they changed behaviour and lost swimming control, moved in circle patterns and splashed in the surface, and some fish even showed an abnormal bending of their body. If these abnormal behaviours arise because of increasing nociception or pain in the skin, possible nociception or pain from organ failure (‘deep pain’)*,* or if they rather reflect neurological and/or physiological impairment and loss of muscle control, or a combination of these, cannot be judge by visual observations alone. However, it can be argued that both temperature itself and possible breakdown of body functions cause nociception or pain, and therefore that the experience of alarm increases with temperature and exposure time.

The struggling behaviour during exposure to warm water would increase the risk for mechanical damage, which may contribute to the relatively high mortality associated with thermal delousing (Overton et al., 2018). In the present study, the threshold for nocifensive or pain behaviour was around 28 °C. However, all individuals were acclimated to 8.5 °C. Acclimation temperature matters for critical temperature for survival, with the critical temperature around 3 °C higher when salmon parr were acclimated to 20 °C than to 5 °C (Elliott, 1991). Anttila et al. (2014) found an even larger effect of acclimation temperature on cardiac capacity. While temperature induced death is usually the result of inner physiological impairment such as cardiac collapse (Anttila et al., 2014), the acute behaviour response is more likely due to nociceptors, and we do not know if the threshold for these nociceptors to respond is affected by acclimation temperature.

**Fig. 5:**
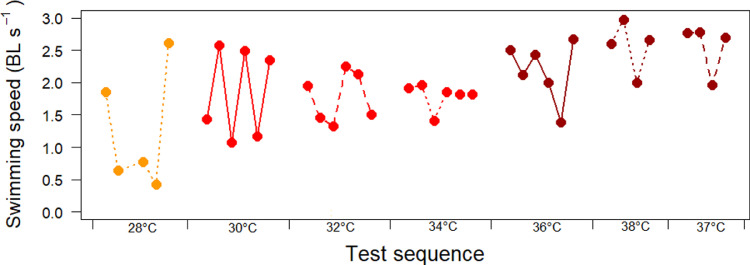
Swimming speed first 10 s for the individual fish in order of test sequence on day 2 of the trial. Point colouring is given for easy identification of temperatures.

A criticism of the study may be that for practical reasons the order of the temperature tests where not randomised, but often incremental due to practical considerations when adding buckets of cooled or heated seawater to regulate water temperature between tests. This meant that water was only changed to a limited degree between fish, and especially between fish tested on the same temperature. However, plotting median initial swimming speed per fish on day 2 of the test procedure with temperature test sequence 28, 30, 32, 34, 36, 38, 37° reveals completely random patterns between fish within the same temperatures (Fig. 5). This supports that the observed behaviours of the fish are responses to changes in temperatures, and not responses to any residues left in the water from the previous fish.

In conclusion, the present study suggests, based on behavioural observations, that temperatures above ~28 °C are acutely aversive to salmon and results in nocifensive or pain responses within seconds.

## Ethical statement

The experiment was conducted in accordance with Norwegian regulations on animal experimentation under permit number 15383.

## Declarations of interest

None

## Declaration of Competing Interest

All authors are employees at independent national research institutes and have no affiliations with or involvement in any organization or entity with any financial or non-financial interest in the subject matter or materials discussed in this manuscript.

## Appendix Supplementary materials

Direction change Image, video 1

Circling Image, video 2

Head shake Image, video 3

Collision Image, video 4

Surface break Image, video 5

Bend and endpoint Image, video 6
